# CIB2 function is distinct from that of whirlin in the organization of sterocilia architecture

**DOI:** 10.1242/dmm.052043

**Published:** 2025-04-03

**Authors:** Arnaud P. J. Giese, Andrew Parker, Sakina Rehman, Steve D. M. Brown, Saima Riazuddin, Craig W. Vander Kooi, Michael R. Bowl, Zubair M. Ahmed

**Affiliations:** ^1^ Department of Otorhinolaryngology – Head and Neck Surgery, University of Maryland School of Medicine, Baltimore, MD 21201, USA; ^2^ MRC Harwell Institute, Mammalian Genetics Unit, Harwell Campus, Oxfordshire OX11 0RD, UK; ^3^ Department of Biochemistry and Molecular Biology, University of Maryland School of Medicine, Baltimore, MD 21205, USA; ^4^ Department of Biochemistry and Molecular Biology, University of Florida, Gainesville, FL 32603, USA; ^5^ UCL Ear Institute, University College London, London WC1X 8EE, UK; ^6^ Department of Ophthalmology and Visual Sciences, University of Maryland School of Medicine, Baltimore, MD 21201, USA; ^7^ Program in Neuroscience and Cognitive Science, University of Maryland, College Park, MD 20742, USA

**Keywords:** CIB2, WHRN, Inner ear hair cells, Stereocilia staircase, Cochlea

## Abstract

Humans and mice with mutations in genes encoding CIB2 and whirlin (WHRN) are deaf. We previously reported that CIB2 binds to WHRN and is essential for stereocilia staircase architecture of cochlear hair cells. Here, we refine the interaction domains of both proteins and show that these proteins play unique roles in stereocilia bundle formation and organization. We found that the EF2 domain of CIB2 binds to the HHD2 region of WHRN. AlphaFold2 multimer independently identified the same interacting regions and gave a thorough structural model. Next, we investigated genetic interaction between murine *Cib2* and *Whrn*. Hearing in mice double heterozygous for functionally null alleles (*Cib2^KO/+^;Whrn^wi/+^*) was similar to that in age-matched wild-type mice, indicating that partial deficiency for both *Cib2* and *Whrn* does not impair hearing. Double homozygous mutant mice (*Cib2^KO/KO^;Whrn^wi/wi^*) were deaf, and their cochlear stereocilia exhibited a predominant phenotype seen in single *Whrn^wi/wi^* mutants. Overexpression of WHRN in *Cib2^KO/KO^* mice did not rescue the stereocilia morphology. These data suggest that CIB2 is multifunctional, with key independent functions in the development and/or maintenance of the stereocilia staircase pattern in auditory hair cells.

## Teaser

**Summary:** Our study reveals that CIB2 is a multifunctional protein with key independent functions in the development of the stereocilia staircase pattern in mouse auditory hair cells.

## INTRODUCTION

Hearing depends upon hair cells, the polarized epithelial cells of the inner ear that have mechano-sensitive hair bundles located at their apical pole. The hair bundle is composed of numerous stereocilia that are organized in a graded staircase pattern. The staircase architecture of the stereocilia bundle is conserved across all vertebrate hair cells and essential for hearing function, because it allows effective pulling of the tip links between stereocilia of neighboring rows. The organization, elongation and row identity of stereocilia in cochlear hair cells are highly regulated and involve several protein complexes, including myosin (MYO)3A, MYO3B (Ebrahim et al., 2016), MYO15A (Belyantseva et al., 2003), whirlin (WHRN) (Belyantseva et al., 2005), epidermal growth factor receptor pathway substrate 8 (EPS8) (Manor et al., 2011; Zampini et al., 2011), EPS8-like 2 (EPS8L2) (Furness et al., 2013) and G protein signaling modulator 2 (GPSM2)/G protein subunit alpha i3 (GNAI3) (Mauriac et al., 2017; Tadenev et al., 2019; Tarchini et al., 2016). For instance, a long isoform of WHRN is localized at the very tips of stereocilia and, together with its carrier – MYO15A, is essential for the normal elongation of stereocilia and formation of the characteristic staircase shape of the hair bundle (Belyantseva et al., 2003, 2005). Furthermore, during development, WHRN targets GPSM2/GNAI3 to the tips of the first-row stereocilia, leading to the accumulation of the elongation protein complex at this site. In the absence of the GPSM2/GNAI3 complex, the first-row stereocilia fail to grow to the correct height, resulting in profound deafness (Mauriac et al., 2017; Tadenev et al., 2019; Tarchini et al., 2016).

We previously reported that, in calcium and integrin binding protein 2 (CIB2) homozygous mutant mice, the overall architecture of the cochlear stereociliary bundle is affected. CIB2 deficiency results in overgrowth of transducing shorter-row stereocilia (rows 2 and 3) in the auditory hair cells, suggesting a direct role of CIB2 in stereocilia staircase patterning (Giese et al., 2017). We have also demonstrated that CIB2 physically interacts with WHRN (Riazuddin et al., 2012). However, our prior studies in *Whrn^wi^* mutant mice revealed that WHRN is not necessary for the localization of CIB2 in mouse inner ear hair cell stereocilia (Riazuddin et al., 2012). Given the role of both CIB2 and WHRN in the normal staircase patterning of stereocilia, and their binding with each other, we sought to determine (1) their interacting domains; (2) whether CIB2 and WHRN have functional overlap with each other; and (3) whether there is genetic interaction between the genes encoding CIB2 and WHRN. To map the interacting regions, we generated a series of CIB2 and WHRN deletion and point mutation fluorescently tagged constructs, and performed nanoscale pulldown (NanoSPD) assays, co-immunoprecipitation studies and molecular modeling. For functional interactions, we adapted classical genetic approaches, and crossed *Cib2^KO^* mice with *Whrn^wi^* (knockout) or *Whrn^BAC279^* (overexpressing) (Mburu et al., 2003) mice and analyzed their first- and second-generation offspring. Analysis of double heterozygotes and double homozygous mutants allowed us to determine whether loss of CIB2 and WHRN affects viability, as both proteins are expressed in many tissues besides inner ear (Riazuddin et al., 2012; Mburu et al., 2003), and produces a more severe inner ear phenotype or a superimposition of pathologies. Analysis of double heterozygotes also allowed us to determine digenic interaction between *Cib2* and *Whrn*. Finally, analysis of offspring from *Cib2^KO/KO^*, *Whrn^BAC279^* crosses was used to determine whether overexpressing WHRN could rescue the stereocilia staircase pathology. Our data suggest that CIB2 has a role that is distinct from that of WHRN in development and/or organization of the stereocilia staircase patterning in the cochlear hair cells.

## RESULTS

### CIB2 is essential for the cochlear hair cell stereocilia staircase pattern

**Fig. 1.**
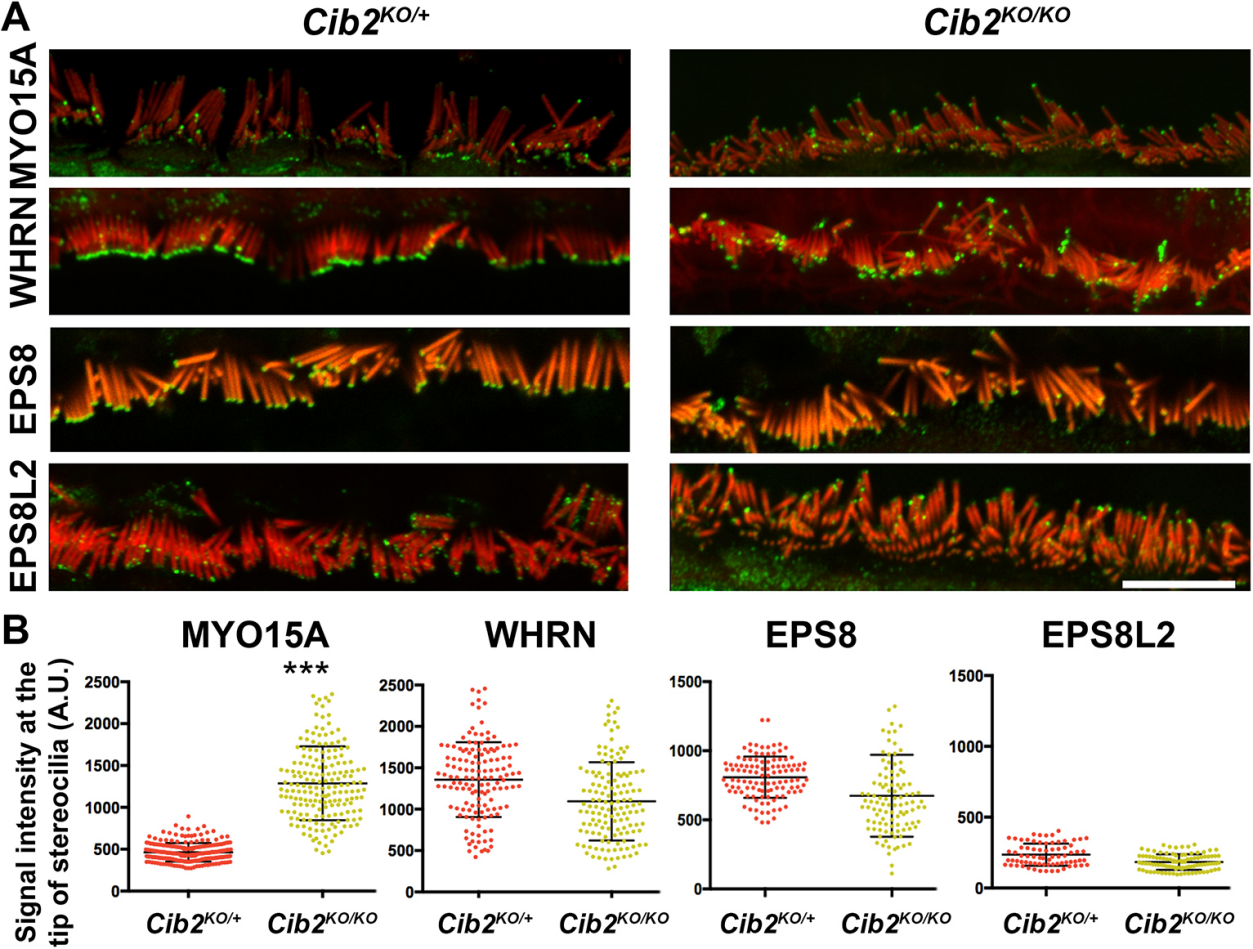
**MYO15A overaccumulates at the tips of stereocilia in *Cib2^KO/KO^* mice.** (A) Expression of MYO15A, WHRN, EPS8 and EPS8L2 proteins in organs of Corti from *Cib2^KO/KO^* mice (right column) and controls (*Cib2^KO/+^* mice, left column) at postnatal day (P)12. In contrast to other proteins, MYO15A showed overaccumulation at the tip of the first row of stereociliary bundle of inner hair cells (IHCs) in *Cib2^KO/KO^* mice. (B) Quantification of fluorescent signal measured by confocal microscopy at the tips of the tallest row of stereocilia, which further confirmed overaccumulation of MYO15A in *Cib2^KO/KO^* mice (***P<0.005, one-way ANOVA). A.U., arbitrary units.

Our previous studies revealed that the row identity of the cochlear stereociliary bundles was not maintained in *Cib2* mutant mice (Giese et al., 2017). Here, we investigated whether the observed loss of row identity is due to a role of CIB2 in the regulation of proteins essential for the staircase pattern (e.g. MYO15A, WHRN, EPS8, etc.) (Belyantseva et al., 2003, 2005; Manor et al., 2011; Zampini et al., 2011). To test this hypothesis, we immunostained organs of Corti from *Cib2* mutant mice (*Cib2^KO/KO^*), along with those from controls, at postnatal day (P)12, for the proteins MYO15A, WHRN, EPS8 and EPS8L2. Immunolabeling using PB48 antibody revealed aberrant staining pattern for MYO15A in *Cib2^KO/KO^* mutants, with overaccumulation at the tips of first-row stereocilia of inner hair cell (IHC) bundles (Fig. 1A). The overaccumulation of MYO15A at the tips of the tallest row of stereocilia was further confirmed by the quantification of fluorescent signal measured using confocal microscopy (Fig. 1B). Furthermore, in the IHCs of *Cib2^KO/KO^* mice, WHRN immunostaining, detected using antibodies specific to the long isoform of WHRN (Belyantseva et al., 2005), was slightly weaker, but not statistically significant, at the tips of stereocilia (Fig. 1A,B). However, EPS8 and EPS8L2 immunostaining in the IHCs of *Cib2^KO/KO^* mice persisted at levels similar to those observed at the tips of stereocilia of control hair cells (Fig. 1A,B).

### CIB2 EF2 binding motif is necessary for CIB2-WHRN interaction

**Fig. 2.**
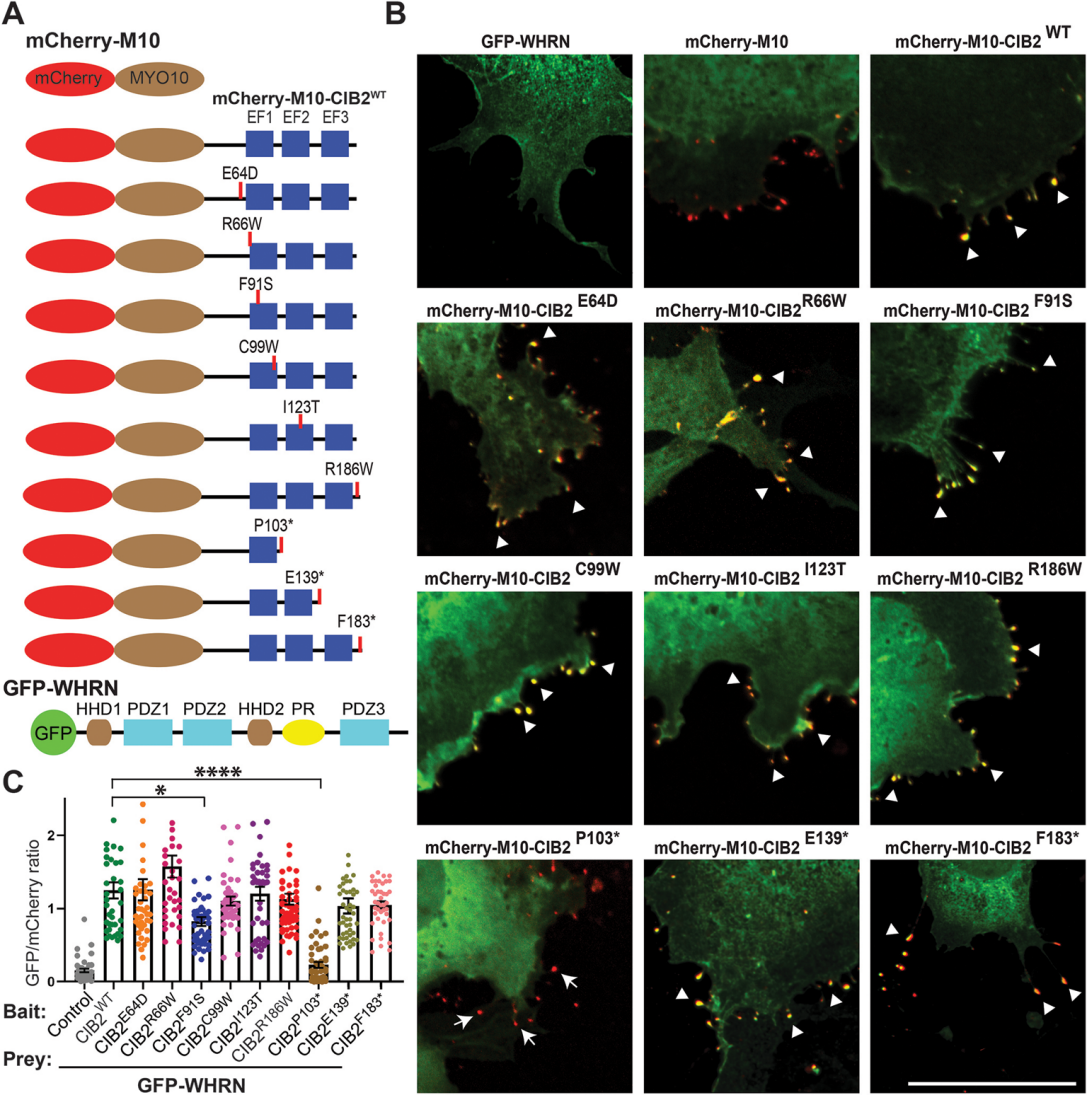
**CIB2 EF2 domain binds WHRN.** (A) Schematic of the mCherry-MYO10, mCherry-MYO10-CIB2^WT^ and CIB2 variants, as well as GFP-WHRN^WT^ construct used for the nanoscale pulldown (NanoSPD) 1.0 assay. (B) COS-7 cells were co-transfected with mCherry-MYO10 or mCherry-MYO10-CIB2 constructs (bait, red) and GFP-WHRN (prey, green), Merge channels are shown; see Fig. S1 for single-channel images. Accumulations of bait and prey at the tips of filopodia are indicated by arrowheads. Arrows indicate the absence of accumulation of prey at the filopodia tip. Scale bar: 10 μm. (C) Quantification of NanoSPD assay results, showing the interaction between WHRN and different CIB2 mutated constructs carrying some pathogenic DFNB48 missense variants, as well as truncations. *P≤0.02; ****P≤0.0001 (one-way ANOVA).

We previously documented that CIB2 directly interacts with WHRN, and forms a tri-partite complex with WHRN and MYO15A (Riazuddin et al., 2012). To further confirm these findings, and to characterize the specific domains required for the CIB-WHRN interaction, we performed NanoSPD assays (Bird et al., 2017). For these studies, COS-7 cells were co-transfected with GFP-WHRN and various mCherry-MYO10-CIB2 deletion constructs (Fig. 2A,B; Fig. S1). The assay, using mCherry-MYO10-CIB2 and full-length GFP-WHRN constructs, confirmed interaction of these proteins. Further, both proteins significantly accumulated at the tip of filopodia in COS-7 cells compared to negative control cells transfected with either mCherry-MYO10 or GFP-WHRN only (Fig. 2B,C).

Next, we performed NanoSPD assays in COS-7 cells using various mCherry-MYO10-CIB2 deafness-causing missense variants (p.Glu64Asp, p.Arg66Trp, p.Phe91Ser, p.Cys99Trp, p.Ile123Thr, p.Arg186Trp) and GFP-WHRN full-length constructs (Fig. 2B,C; Fig. S1). Except the p.Phe91Ser variant, none of the other tested missense pathogenic variants affect the CIB2-WHRN complex (Fig. 2C). Although the interaction between CIB2, harboring p.Phe91Ser variant, and WHRN was maintained, it was significantly weaker (*P=0.0247) than that between WHRN and wild-type (WT) CIB2 (Fig. 2C). Next, as bait, we used several truncated CIB2 constructs deleting the EF hand domains (p.Pro103*, p.Gln139*, p.Phe183*). The p.Pro103* truncation completely abolished the WHRN interaction, whereas p.Gln139* and p.Phe183* truncated CIB2 proteins retained the ability to bind WHRN (Fig. 2B,C).

### WHRN HDD2 domain is required for interaction with CIB2

**Fig. 3.**
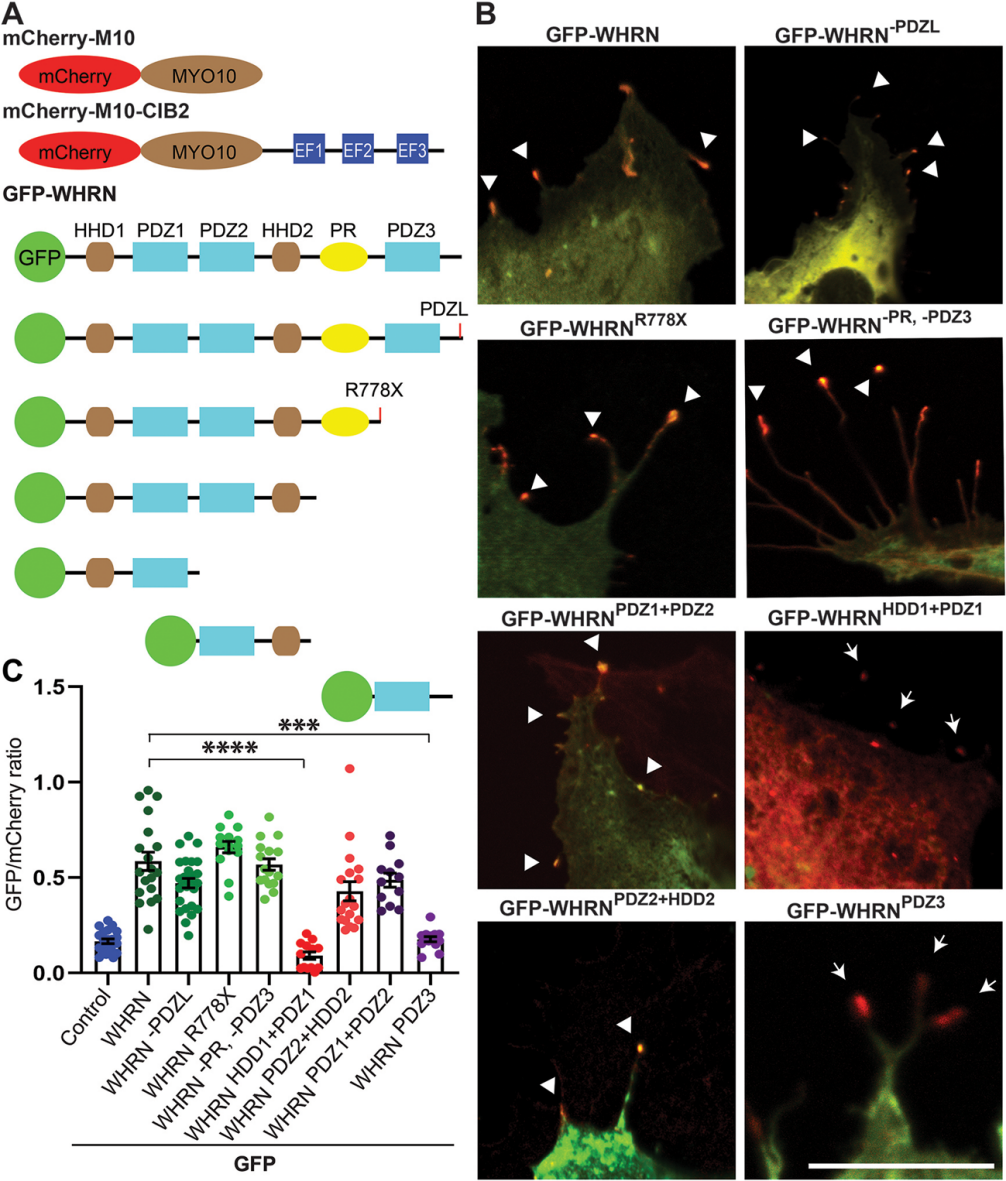
**WHRN PDZ2-HDD2 regions bind CIB2.** (A) Schematic of the mCherry-MYO10-CIB2^WT^ and GFP-WHRN^WT^ and deletion constructs used for the NanoSPD 1.0 assay. (B) COS-7 cells were co-transfected with mCherry-MYO10-CIB2^WT^ (bait, red) and GFP-WHRN constructs (prey, green), Merge channels are shown; see Fig. S2 for single-channel images. Accumulations of bait and prey at the tips of filopodia are indicated by arrowheads. Arrows indicate the absence of accumulation of prey at the filopodia tip. Scale bar: 10 μm. (C) Quantification of NanoSPD assay results, showing the interaction between WHRN PDZ2-HDD2 region-containing constructs and CIB2. ***P≤0.001; ****P≤0.0001 (one-way ANOVA).

We next investigated the specific domain region within WHRN required for interaction with CIB2. We performed NanoSPD assay in COS-7 cells using full-length mCherry-MYO10-CIB2 and GFP-WHRN truncated constructs (Fig. 3; Fig. S2). All the GFP-WHRN truncated constructs that have PDZ2 and HDD2 domains in them co-accumulated at the tips of filopodia, suggesting that the PDZ1 and PDZ3 domains are not necessary for CIB2-WHRN interaction (Fig. 3B,C; Fig. S2).

**Fig. 4.**
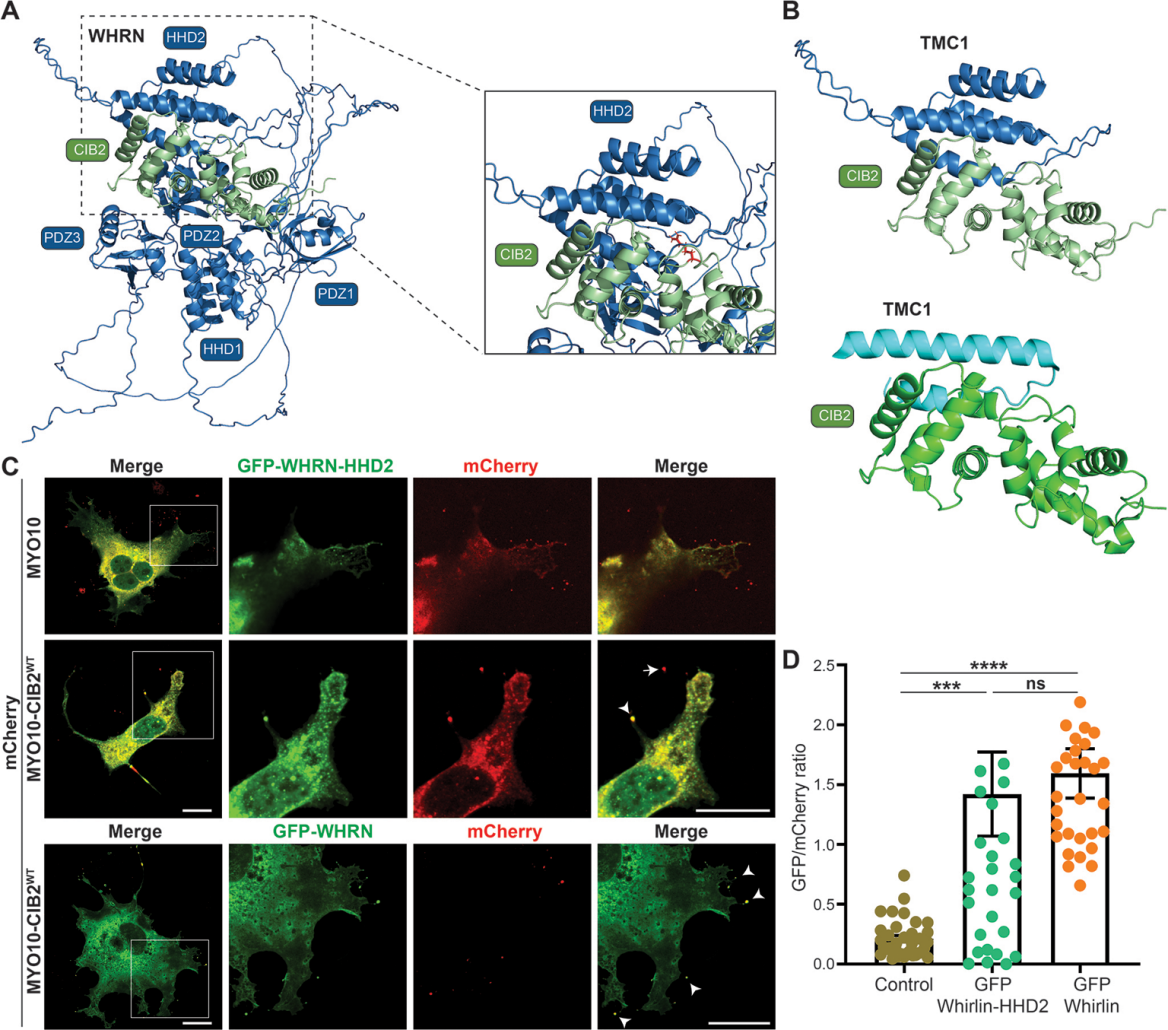
**WHRN HDD2 region binds CIB2 EF2 region.** (A) AlphaFold2 multimer model of the complex between WHRN (blue) and CIB2 (green). (B) Zoom of the predicted specific interacting HHD2 region of CIB2 (top) and comparison to the known structure of TMC1 bound to CIB2 (bottom). (C) COS-7 cells were co-transfected with mCherry-MYO10-CIB2^WT^ (bait, red) and GFP-WHRN-HDD2 construct (prey, green). Merge channels for the whole cell, and single and merged channels for zoom-in (boxed) regions are shown. Arrow indicates CIB2 alone. Arrowheads indicate WHRN and CIB2 colocalization. Scale bars: 10 μm. (D) Quantification of NanoSPD assay results, showing the interaction between WHRN PDZ2-HDD2 region and CIB2. n.s., non-significant; ***P≤0.001; ****P≤0.0001 (one-way ANOVA).

To gain detailed insight into the interacting regions of CIB2-WHRN, we generated an AlphaFold2 multimer (Jumper et al., 2021; Tunyasuvunakool et al., 2021) model of the CIB2 in complex with WHRN (Fig. 4A,B; Fig. S3). Significant complex formation was predicted between CIB2 and well-defined regions of WHRN, involving 83 and 74 residues, respectively, burying 2693 Å (Belyantseva et al., 2003). The predicted interaction is between the CIB2 C-terminal EF2 domain and the HDD2 domain of WHRN (Fig. 4A). The involvement of the CIB2 C-terminal domain is consistent with deletion experiments, discussed above. Further, analysis of the complex indicated that it was strongly centered on the HDD2 domain of WHRN, with eight of ten potential salt bridges, and 12 of 16 potential hydrogen bonds, contributed by the HDD2 domain of WHRN (Fig. S3). Strikingly, this potential interaction is highly structurally homologous to that formed between the high-affinity transmembrane channel-like protein 1 (TMC1)-binding region and CIB2 (Liang et al., 2021; Giese et al., 2025) (Fig. 4B). Indeed, the core helical CIB2-binding regions of both TMC1 and WHRN have shared highly hydrophobic faces bound to the EF2 domain of CIB2, suggesting a competitive interaction.

Based on these predictions, we generated a GFP-WHRN-HDD2 domain-only construct (NM_001008791.2; residues 415-561) and tested its interaction with full-length CIB2 through NanoSPD assay (Fig. 4C,D). Consistent with the AlphaFold2 prediction, CIB2 interaction with the HDD2 region was comparable to that with full-length WHRN protein (Fig. 4C,D). Taken together, these data establish that the CIB2-WHRN interaction is mediated through the EF2 domain of CIB2 and the HDD2 domain of WHRN.

### Altering WHRN levels does not restore normal stereocilia architecture in *Cib2^KO/KO^* mice

**Fig. 5.**
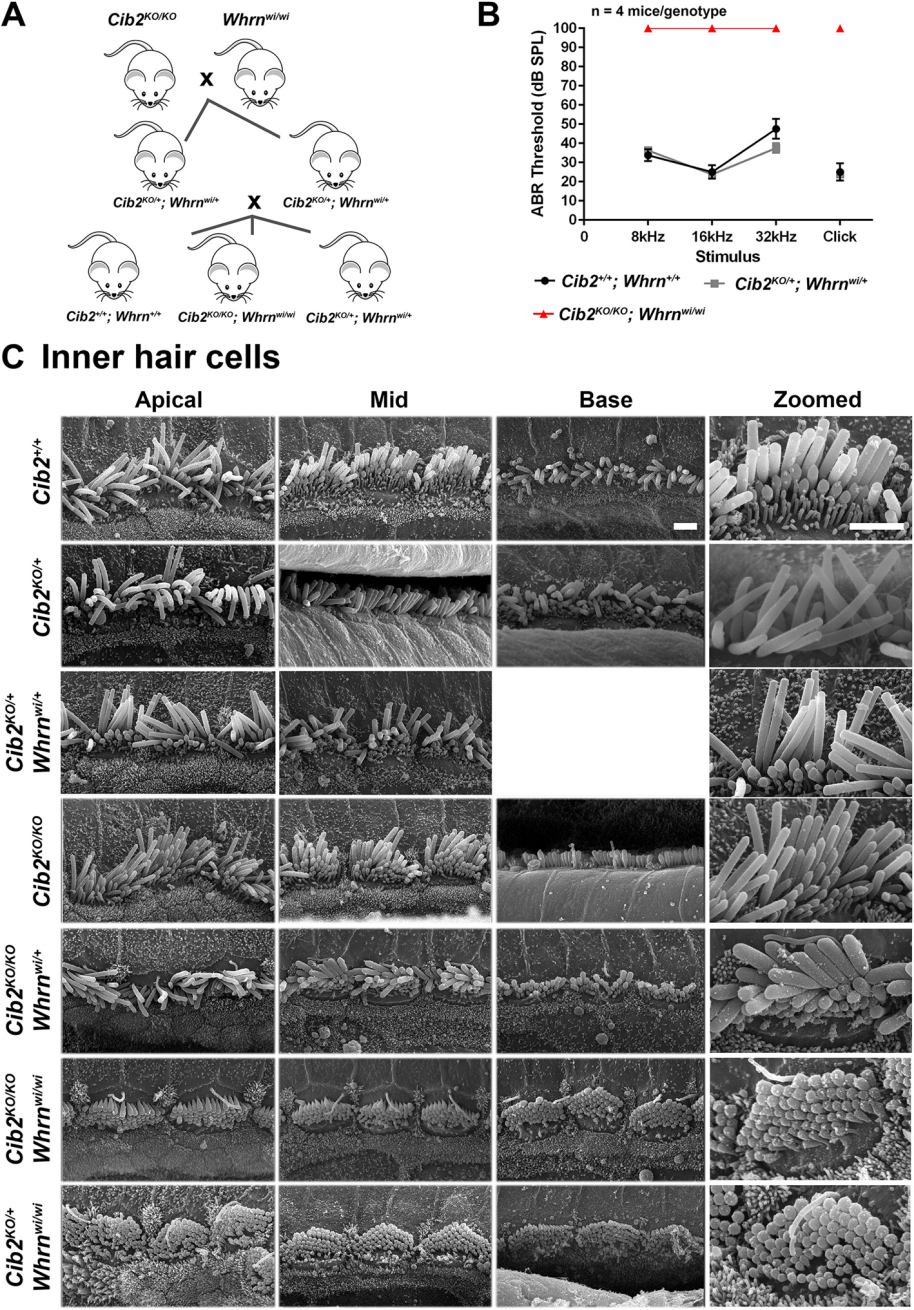
**No genetic interactions between *Cib2* and *Whrn* result in hearing loss or defects in hair cell bundle morphology.** (A) Cartoon showing the double mutant intercross breeding strategy that was employed to obtain the genotypes required for the study. Only desired genotypes are shown. (B) Audiogram showing the auditory brainstem response (ABR) thresholds of 12- to 16-week-old mice. The data show that *Cib2^KO/+^;Whrn^wi/+^* mice (*n*=4) have similar thresholds to *Cib2^+/+^;Whrn^+/+^* mice (*n*=4), suggesting that both *Cib2* and *Whrn* are haplosufficient. As expected from ABR thresholds reported in mice homozygous for either mutation, the double homozygous mutant *Cib2^KO/KO^;Whrn^wi/wi^* mice (*n*=3) exhibited no response to the highest dB stimulus at any frequency tested. Data shown are mean ABR thresholds±s.e.m. SPL, sound pressure level. (C) Scanning electron micrographs of IHCs from 2-week-old *Cib2;Whrn* mice. Representative scanning electron micrographs of IHC bundles from the apical, mid and basal cochlear turns of 2-week-old mice. *Cib2^+/+^*, heterozygous *Cib2^KO/+^* and *Cib2^KO/+^*;*Whrn^wi/+^* double heterozygous mice have bundles that are very similar in appearance. Interestingly, in homozygous *Cib2^KO/KO^* mice, IHC bundles still have kinocilia present across all turns. This developmental structure usually retracts during the first week postpartum. Moreover, additional rows of stereocilia are present compared with *Cib2^+/+^* and *Cib2^KO/+^* mice. IHC bundles of *Cib2^KO/KO^*;*Whrn^wi/+^* mice show no obvious difference from those of *Cib2^KO/KO^* mice, indicating that WHRN haploinsufficiency does not overtly potentiate the *Cib2* null phenotype. IHC bundles of *Cib2^KO/KO^*;*Whrn^wi/wi^* mice display short stereocilia, additional rows of stereocilia and kinocilia in all turns. These are features observed in both *Whrn^wi/wi^* and *Cib2^KO/KO^* mutants. IHC bundles of *Cib2^KO/+^;Whrn^wi/wi^* mice have very short stereocilia, and the kinocilia is still present in the apical turn; these findings are in agreement with published findings on *Whrn^wi/wi^* (Holme et al., 2002) where, in some cases, persistence of kinocilia has been noted. *n*≥3 for each genotype. Scale bars: 2 µm.

Given the interaction between CIB2 and WHRN, increased expression of MYO15A (WHRN transporter) in *Cib2* mutant mice, and the role of both proteins in orchestrating the staircase pattern of stereocilia bundle, we next sought to determine whether altered WHRN levels are responsible for impaired stereocilia architecture in *Cib2^KO/KO^* mice using a classical genetic approach. To test this, we crossed *Cib2^KO/KO^* and *Whrn^wi/wi^* mutants (Fig. 5A) and analyzed first- and second-generation offspring. Analysis of double heterozygous (*Cib2^KO/+^;Whrn^wi/+^*) and double mutants (*Cib2^KO/KO^;Whrn^wi/wi^*) allowed us to determine whether deficiencies in, or loss of, CIB2 and WHRN produces a more severe phenotype or a superimposition of pathologies.

First, WT, double heterozygous and double homozygous mutants from these crosses were subjected to auditory brainstem response (ABR) measurements at 12-16 weeks of age (Fig. 5B). At this age, the double heterozygous *Cib2^KO/+^;Whrn^wi/+^* mice had ABR thresholds similar to those of their WT littermates (Fig. 5B). In contrast to WT and double heterozygous mice, as anticipated from the reported phenotypes of *Cib2^KO/KO^* and *Whrn^wi/wi^* mice (Giese et al., 2017), the double mutants *Cib2^KO/KO^;Whrn^wi/wi^* had no response to any sound stimuli (Fig. 5B).

**Fig. 6.**
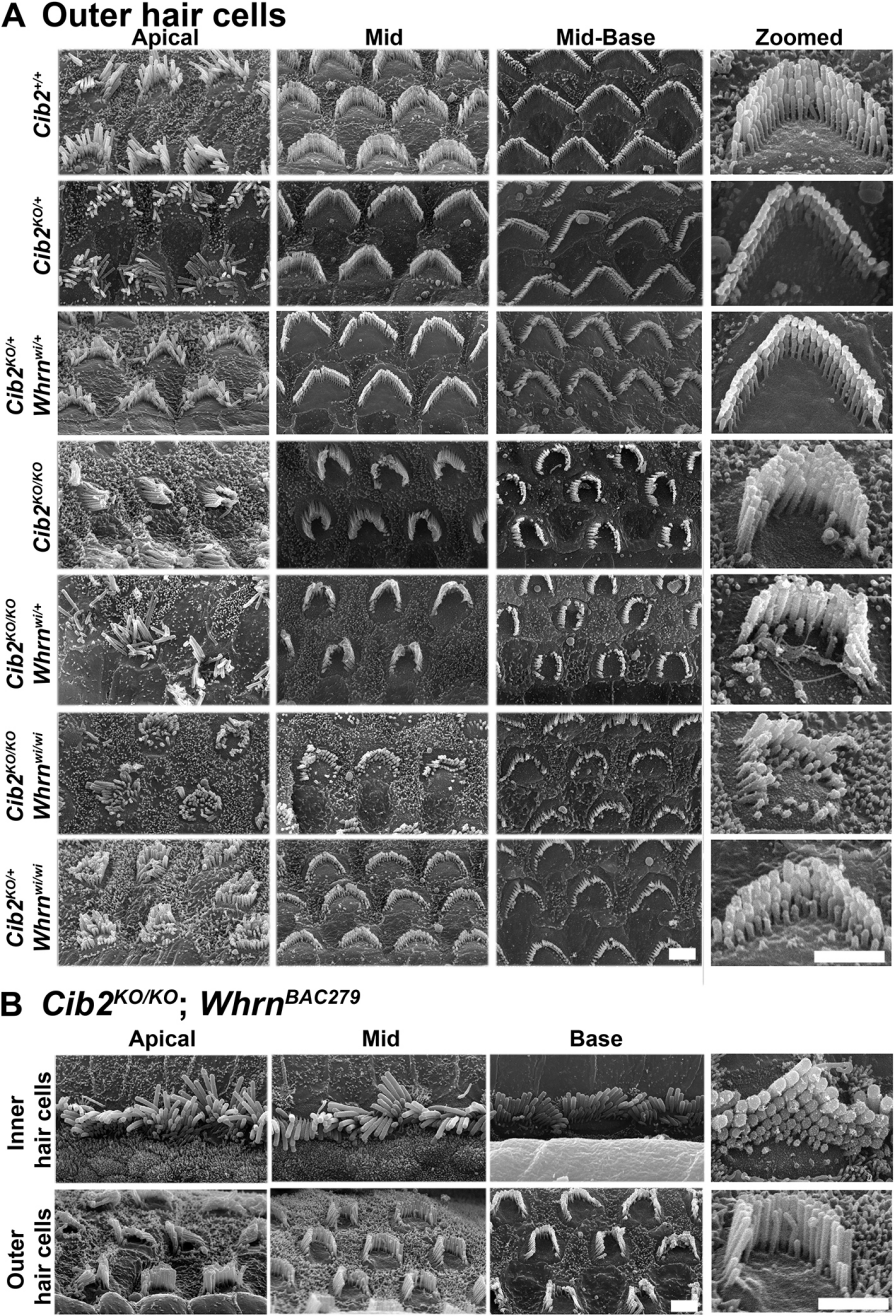
**Overexpressing WHRN fails to restore stereocilia staircase pattern in *Cib2^KO/KO^* mice.** (A) Representative scanning electron micrographs of outer hair cell (OHC) bundles from the apical, mid and basal cochlear turns of 2-week-old mice. *Cib2^+/+^*, heterozygous *Cib2^KO/+^* and *Cib2^KO/+^*;*Whrn^wi/+^* double heterozygous mice have bundles that are very similar in appearance. OHC bundles of *Cib2^KO/KO^* mice are poorly developed, displaying a crescent shape rather than the usual W-shape formation, and the staircase is poorly defined. Similar to IHC bundles, OHC bundles of *Cib2^KO/KO^*;*Whrn^wi/+^* mice show no obvious difference from those of *Cib2^KO/KO^* mice, indicating that WHRN haploinsufficiency does not overtly potentiate the *Cib2* null phenotype. However, OHC bundles of *Cib2^KO/KO^;Whrn^wi/wi^* and *Cib2^KO/+^;Whrn^wi/wi^* mice are very poorly developed. *n*≥3 for each genotype. (B) Representative scanning electron micrographs of IHC and OHC bundles from the apical, mid and basal cochlear turns of 2-week-old *Cib2^KO/KO^*;*Whrn^BAC279^* mice. The shape and appearance of IHC and OHC bundles appear grossly similar to those of *Cib2^KO/KO^* mice, indicating that overexpression of WHRN does not affect the *Cib2* null phenotype. *n*≥3 for each genotype. Scale bars: 2 µm.

Next, we examined by scanning electron microscopy (SEM) the inner ears of double mutants and controls at 2 weeks of age. SEM images from all the double mutant mice exhibited an apparent phenotype (Fig. 5C and Fig. 6A) of *Whrn^wi/wi^* mutants (Holme et al., 2002) with some superimposition of features of *Cib2^KO/KO^* mice (Giese et al., 2017). *Whrn* mutants had extremely short stereocilia in IHCs and outer hair cells (OHCs) (Holme et al., 2002). In contrast, the OHCs in *Cib2^KO/KO^* mutant mice often had overgrowth of the second row of stereocilia and horseshoe-shape bundle, whereas the IHCs had abnormally thick third- and fourth-row stereocilia and persistent kinocilia (Giese et al., 2017). The *Cib2^KO/KO^;Whrn^wi/wi^* double mutant mice had shorter stereocilia bundle, with kinocilia failing to regress properly (Fig. 5C and Fig. 6A). Moreover, reducing the levels of either protein (CIB2 or WHRN) (Wang et al., 2017) on the genetic background of the other mutant strain (*Cib2^KO/KO^;Whrn^wi/+^* or *Cib2^KO/+^;Whrn^wi/wi^*) neither worsened nor rescued the normal staircase pattern in either situation (Fig. 5C and Fig. 6A). A prior study reported reduced levels of WHRN in the shaft of stereocilia of auditory hair cells in *Cib2^KO/KO^* mice (Michel et al., 2017). Therefore, we also investigated the impact of WHRN overexpression in *Cib2^KO/KO^* mice. For these studies, we crossed and generated mice that were homozygous for the *Cib2^KO^* allele and were also positive for the *Whrn^BAC279^* transgene (Mburu et al., 2003). Overexpressing WHRN, using the *Whrn^BAC279^* strain, also failed to restore the stereocilia staircase pattern in *Cib2^KO/KO^* mice (Fig. 6B). Collectively, these results support the notion that CIB2 and WHRN proteins have coordinated, but non-overlapping, functions in orchestrating the stereocilia staircase pattern and bundle shape.

## DISCUSSION

In this study, we demonstrate that CIB2 is multifunctional, with key independent functions in development and/or maintenance of stereocilia staircase pattern in auditory hair cells. Although, we cannot rule out the possibility of lack of interaction due to the misfolding of the deletion constructs (e.g. GFP-WHRN HHD1-PDZ1), both our NanoSPD-based interaction assays and AlphaFold2 multimer (Jumper et al., 2021) prediction model support the interaction between the CIB2 C-terminal EF2 domain and the HDD2 domain of WHRN (Fig. 4A).

Loss of CIB2 caused a slight, but not statistically significant, reduction in WHRN levels at the tips of the tallest rows of IHC stereocilia. However, double heterozygous mice from a *Cib2^KO^*×*Whrn^wi^* cross exhibited normal startle responses to sound (Fig. 5B). The double homozygous mutants of *Cib2* and *Whrn* exhibited profound hearing loss. The morphology of cochlear hair cell stereocilia in double homozygous mutant mice suggests a superimposition of the phenotypes generated by each of the single homozygotes. Non-overlapping functions would be expected to generate a more pronounced phenotype. Furthermore, overexpression of WHRN in *Cib2^KO^* mice did not restore normal staircase architecture of stereocilia in cochlear hair cells. Taken together, our studies indicate that CIB2 performs a distinct function in regulating the staircase architecture of cochlear hair cell stereocilia that does not obviously overlap with the function of WHRN. The superimposition of phenotypes in the double homozygous mutant mice indicates that CIB2 and WHRN have unique and specific functions in stereocilia bundle development and patterning. This is consistent with molecular analysis, suggesting overlapping mutually exclusive binding interfaces on CIB2 for WHRN and TMC1. The nature of the formed complexes will be governed by inherent binding affinities, the stoichiometry of components, and the complex nature of interactions involving multiple dynamic molecular components. These factors warrant further investigation.

Based upon the present study, and upon the individual differences in the stereocilia bundle phenotypes of *Cib2* and *Whrn* single homozygous mutant mice (Giese et al., 2017), both *Cib2* and *Whrn* appear to play distinct roles in establishing the correct architecture of stereocilia bundles. Recent studies have also demonstrated a critical role of MET activity in regulating the staircase pattern of stereocilia bundles in developing cochlear hair cells (Krey et al., 2020). In Usher mutant mouse models, such as *whirler* (*Whrn^wi/wi^*), *shaker* (*Myo7a^sh1/sh1^*), *Ush1g* (*Ush1g^js/js^*) and *Ush1c* (*Ush1c^dfcr/dfcr^*), in which MET is abolished in sensory hair cells, it has been reported that the stereocilia staircase pattern is altered, and that stereocilia are dramatically reduced in length, suggesting that the MET machinery has a positive effect on F-actin polymerization (Krey et al., 2020; Velez-Ortega et al., 2017).

Several studies have reported loss of MET function in *Cib2* mutant mice (Giese et al., 2017; Liang et al., 2021; Michel et al., 2017). However, in *Cib2* mutants, the second- and third-row stereocilia are elongated, which is opposite to the expected retraction of transducing stereocilia that occurs after loss of MET current (Krey et al., 2020; Velez-Ortega et al., 2017). Thus, CIB2 likely has a role in stereocilia growth, unrelated to MET. Recent studies demonstrated that GPSM2 and inhibitory G proteins of the alpha family (GNAI1, GNAI2 and GNAI3) form a complex, which is essential for stereocilia elongation and organization into a staircase pattern (Mauriac et al., 2017; Tadenev et al., 2019). GPSM2-GNAI binds to WHRN, and the whole complex relies on MYO15A to be transported to the tips of stereocilia (Mauriac et al., 2017; Tadenev et al., 2019). As hair cells mature, the GPSM2-GNAI complex and its partners are trafficked to the tips of stereocilia adjacent to the bare zone by the MYO15A motor, thereby establishing the ‘identity’ of the first, tallest row of stereocilia (Tadenev et al., 2019). Bearing in mind the abnormal stereocilia heights in *Cib2* mutants, it could be speculated that CIB2 has a role in the GPSM2-GNAI stereocilia elongation complex.

## MATERIALS AND METHODS

### Animals

All animal procedures were approved by the Institutional Animal Care and Use Committees (IACUCs) of the participating institutes. Animal strains used in this study have been previously reported (Giese et al., 2017; Mburu et al., 2003).

### Immunostaining and confocal imaging

The cochlear and vestibular sensory epithelia were isolated, fine dissected and permeabilized in 0.25% Triton X-100 for 1 h, and blocked with 10% normal goat serum in PBS for 1 h. The tissue samples were probed with primary antibodies against MYO15A, WHRN, EPS8 or EPS8L2 overnight, and, after three washes, they were incubated with the secondary antibody for 45 min at room temperature (RT). Rhodamine phalloidin or Alexa Fluor phalloidin 488 were used at a 1:250 dilution for F-actin labeling. Nuclei were stained with DAPI (Molecular Probes). Images were acquired using either a LSM 700 laser scanning confocal microscope (Zeiss) with a 63×/1.4 NA or 100×/1.4 NA oil immersion objectives or a Leica SP8 laser scanning confocal microscope with a 100×/1.44 NA objective. Stacks of confocal images were acquired with a *z*-step of 0.05-0.5 µm and processed using ImageJ software [National Institutes of Health (NIH)]. Experiments were repeated at least three times, using at least three different animals.

### *CIB2* and *WHRN* constructs and plasmids

Human full-length *CIB2* and *WHRN* cDNA constructs were generated as previously described (Riazuddin et al., 2012). Site-directed mutagenesis was performed on the full-length constructs using QuickChange PCR (Stratagene) to generate specific truncated or mutated versions. All constructs were sequence verified before use in the experiments.

### NanoSPD assay

For NanoSPD assays, we followed the instructions reported previously (Bird et al., 2017; Sethna et al., 2021). Briefly, 60-70% confluent COS-7 cells in six-well plates for nanoTRAP [a construct consisting of GFP nanobody fused with the heavy meromyosin (HMM domain) of myosin 10 (myo10HMM-GFP nanobody); this allows preferential migration of any GFP-tagged protein to the filopodia tips in the transfected cells] were transfected with Lipofectamine 2000 (3:1 ratio) with 1 µg plasmid construct each (nanoTRAP, GFP-tagged bait and prey), and, 24 h post-transfection, cells were split 1:10 ratio on glass coverslips to allow for filopodia formation. The following day, cells were fixed with 4% paraformaldehyde for 15 min at RT and permeabilized with 0.2% Triton X-100 in PBS for 15 min at RT, followed by blocking with 10% normal goat serum (NGS) in PBS for at least 30 min at RT. Primary antibodies were diluted (1:200) in 3% NGS-PBS and incubated overnight at 4°C, followed by the incubations with the indicated goat secondary antibodies (diluted 1:1000). A Zeiss 710 laser scanning confocal microscope or Nikon W1 spinning disk microscope was used for image acquisition.

Quantification of fluorescence intensities at the tips of the filopodia was performed using ImageJ software (NIH). Each value is an average over a square area of 1 μm^2^, with a center at the tip of each individual filopodium (*n*≥60). Data are expressed as a ratio of GFP/mCherry intensities. Each value was then normalized against the values measured in the cells transfected with the bait constructs only. All data represent the mean±s.e.m. One-way ANOVA with Tukey's multiple comparisons test was used to compare the different groups of independent samples.

### AlphaFold

An AlphaFold2 (Jumper et al., 2021) multimer model (Evans et al., 2021 preprint) of the CIB2/WHRN complex was generated using the Colab server without template constraint (https://github.com/sokrypton/ColabFold). Interaction interfaces were analyzed using PDBePISA (Krissinel and Henrick, 2007). Structural models were analyzed, and figures were prepared, using PyMOL (Version 2.4.1, Schrödinger).

### ABRs

Hearing thresholds of mice at 12-16 weeks of age (*n*=4/genotype) were evaluated by recording ABR. All ABR recordings, including broadband clicks and tone-burst stimuli at three frequencies (8, 16 and 32 kHz), were performed using an auditory-evoked potential RZ6-based auditory workstation (Tucker-Davis Technologies) with high-frequency transducer RA4PA Medusa PreAmps. Maximum sound intensity tested was 100 dB sound pressure level. TDT system III hardware and BioSigRZ software (Tucker Davis Technology) were used for stimulus presentation and response averaging.

### SEM

*C*ochleae were fixed in 2.5% glutaraldehyde in 0.1 M cacodylate buffer, pH 7.4 (Electron Microscopy Sciences, Hatfield, PA, USA) supplemented with 2 mM CaCl_2_ (Sigma-Aldrich) for 1-2 h at RT. Then, the sensory epithelia were dissected in distilled water, dehydrated through a graded series of ethanol, critical point dried from liquid CO_2_ (Leica EM CPD300), sputter coated with 5 nm platinum (Q150T, Quorum Technologies, Guelph, Canada), and imaged with a field-emission scanning electron microscope (Helios Nanolab 660, FEI, Hillsboro, OR, USA).

## Supplementary Material

10.1242/dmm.052043_sup1 Supplementary information
