# Supplementary material for: CIB2 function is distinct from that of whirlin in the organization of sterocilia architecture
Source: Dis Model Mech. 2025 Apr 3;18(3):dmm052043. doi: 10.1242/dmm.052043 (PMC11992350; doi:10.1242/dmm.052043)
Supplement: Supplementary information [file dmm-18-052043-s1.pdf]

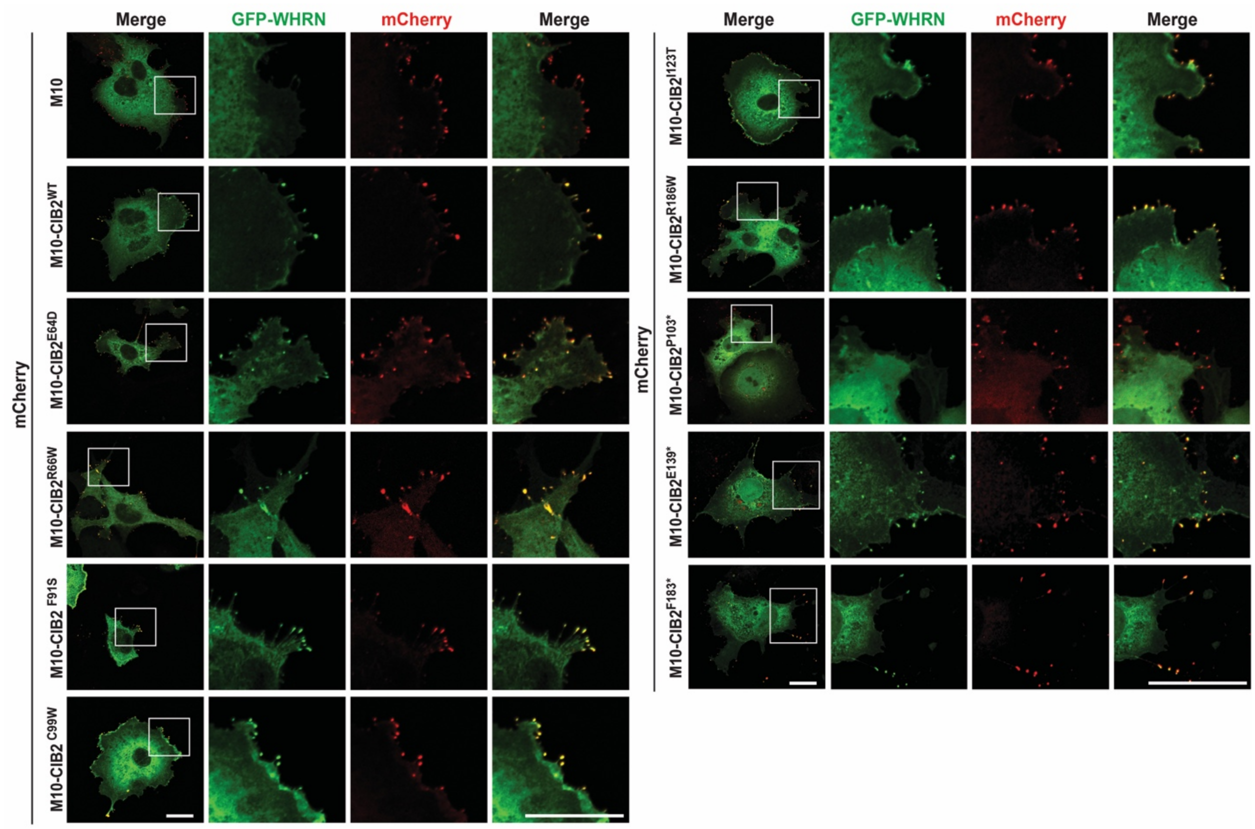

Fig. S1. Nanoscale pull down using CIB2 variants and whirlin^{WT} constructs

COS-7 cells were co-transfected with mCherry-myo10-CIB2 variant constructs (Baits, red) and GFP-whirlin truncated constructs (Prey, green). Single channels are shown. mCherry-myo10 construct was used as a negative control. Scale bar = 10 µm.

Disease Models & Mechanisms • Supplementary information

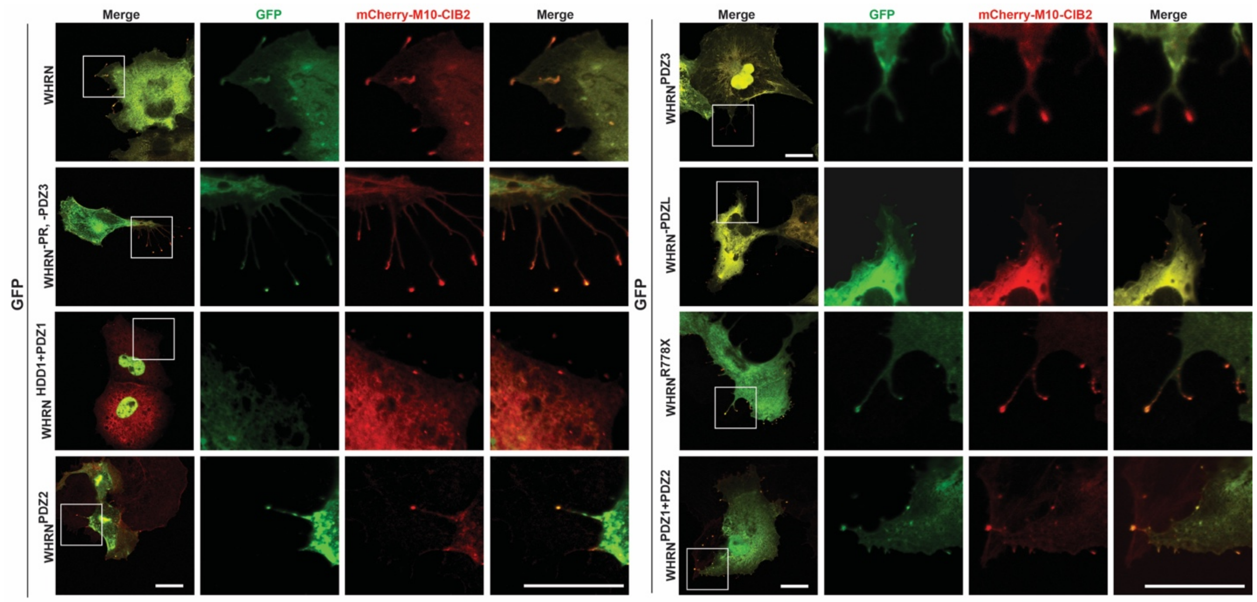

Fig. S2. Nanoscale pull down using CIB2 and truncated whirlin constructs

COS-7 cells were co-transfected with mCherry-myo10-CIB2^{WT} constructs (Baits, red) and GFP-whirlin truncated constructs (Prey, green). Single channels are shown. Scale bar = 10 µm.

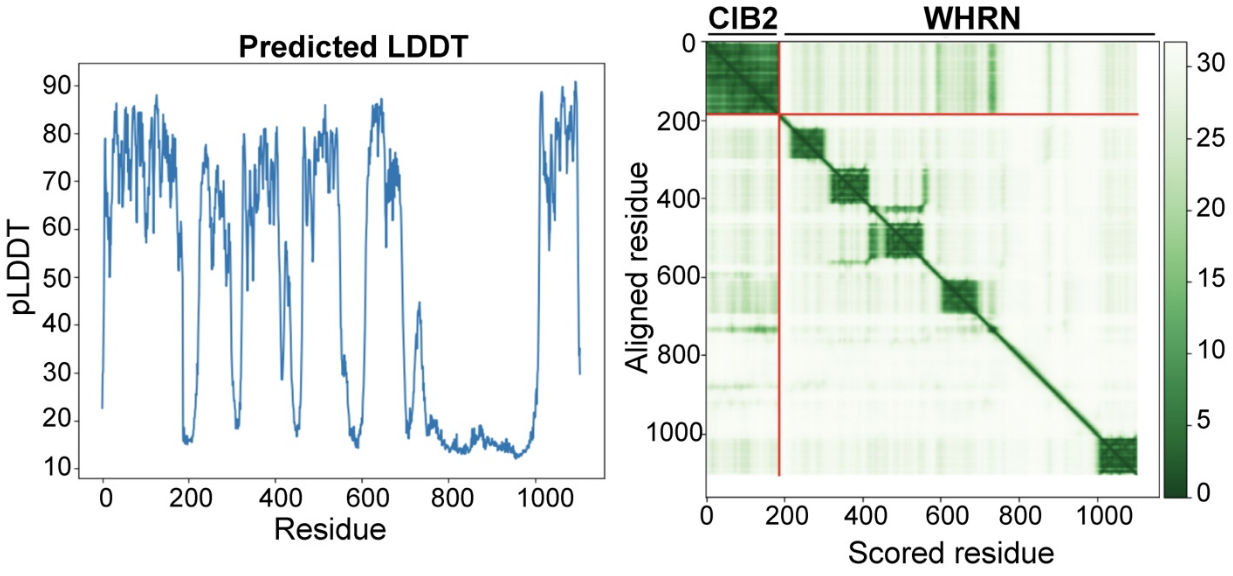

Fig. S3. **Alphafold multimer prediction quality measures for the CIB2-whirlin complex, including predicted Local Distance Difference Test (pLDDT) (left) and Predicted Aligned Error (PAE) (right).**

Disease Models & Mechanisms • Supplementary information
